# Robust iterative closest point algorithm based on global reference point for rotation invariant registration

**DOI:** 10.1371/journal.pone.0188039

**Published:** 2017-11-27

**Authors:** Shaoyi Du, Yiting Xu, Teng Wan, Huaizhong Hu, Sirui Zhang, Guanglin Xu, Xuetao Zhang

**Affiliations:** 1 School of Electronic and Information Engineering, Xi’an Jiaotong University, Xi'an, Shaanxi Province, P.R., China; 2 School of Software Engineering, Xi’an Jiaotong University, Xi'an, Shaanxi Province, P.R., China; Chinese Academy of Sciences, CHINA

## Abstract

The iterative closest point (ICP) algorithm is efficient and accurate for rigid registration but it needs the good initial parameters. It is easily failed when the rotation angle between two point sets is large. To deal with this problem, a new objective function is proposed by introducing a rotation invariant feature based on the Euclidean distance between each point and a global reference point, where the global reference point is a rotation invariant. After that, this optimization problem is solved by a variant of ICP algorithm, which is an iterative method. Firstly, the accurate correspondence is established by using the weighted rotation invariant feature distance and position distance together. Secondly, the rigid transformation is solved by the singular value decomposition method. Thirdly, the weight is adjusted to control the relative contribution of the positions and features. Finally this new algorithm accomplishes the registration by a coarse-to-fine way whatever the initial rotation angle is, which is demonstrated to converge monotonically. The experimental results validate that the proposed algorithm is more accurate and robust compared with the original ICP algorithm.

## Introduction

Point set registration has become an important topic of computer vision and pattern recognition due to its wide applications such as 3D reconstruction [1,2], medical image analysis [3,4], and image retrieval and classification [5,6]. Of many point set registration methods in existence, the iterative closest point (ICP) algorithm [7–9] has received significant attention because of its simplicity and efficiency.

In recent years, a number of researchers have devoted to the improvement of ICP, especially the speed and the robustness. To speed up the computation, a combination of ICP variants was proposed, but it needed a good initial guess [10]. Fitzgibbon proposed the Levenberg-Marquardt algorithm to speed up ICP [11]. In addition, many scholars made efforts to enhance the robustness. Lee et al. introduced a reliability matrix to present the rotation components of ICP [12]. Granger and Pennec proposed a multi-scale schedule using simulated annealing (SA) algorithm to strengthen the robustness [13].

Meanwhile, point set registration with the outliers and noises has drawn the attention of many researchers. Chetverikov et al. presented the trimmed ICP (TrICP) for partial registration by incorporating an overlapping percentage into a least square function [14]. Ridene and Goulette proposed a method using an adaptive dynamic threshold along with the RANSAC to remove outliers [15]. Du et al. proposed the probability ICP, aiming at solving the registration of point sets with noises [16].

However, the initial values problem remained to be solved in the above mentioned approaches. The importance of initial values lies in that an inappropriate initial value could lead ICP algorithm to trap in a local minimum. For instance, when the rotation angle between the two point sets is large and the initial guess is not accurate, the algorithm cannot register point sets correctly. To cope with this problem, we introduce a global reference point. Based on this, we can obtain the relatively accurate initial parameters by making use of the invariance of the distance between each point of sets and the global reference point. And then the point set registration problem can be solved by the new ICP algorithm.

This paper is organized as follows. In section 2, a brief review of the original ICP algorithm is stated. In section 3, aiming at solving the initial parameters of ICP algorithm, a global reference point is introduced and a robust ICP algorithm for large-angle rotation point set registration is proposed. In section 4, experimental results on part B of CE-Shape-1 are shown. In the last section, a conclusion is given finally.

## The ICP algorithm

Given two *n*-dimension point sets in ℝ^*n*^, the shape point set $P≜{\left\{{\vec{p}}_{i}\right\}}_{i=1}^{N_{p}}(N_{p}\in N)$ and the model point set $M≜{\left\{{\vec{m}}_{j}\right\}}_{j=1}^{N_{m}}(N_{m}\in N)$. The objective of original ICP algorithm is to find a rigid transformation, with which the shape point set is in the best alignment with the model point set. For the registration of these two point sets, the formulation is based on the least squares (LS) criterion as follows:

$$\begin{array}{c} \underset{R,\vec{t},c(i)\in \left\{1,2,\cdots ,N_{m}\right\}}{\min}\sum_{i=1}^{N_{p}}\|(R{\vec{p}}_{i}+\vec{t})-{\vec{m}}_{c(i)}{\|}_{2}^{2}\\ s.t.\;R^{T}R=I_{n},\det(R)=1 \end{array}$$ (1)

where R ∈ ℝ^*n*×*n*^ is a rotation matrix, and $\vec{t}\in R^{n}$ is a translation vector.

The standard ICP algorithm solves this problem by iteratively registering the shape point set to the model point set with rotation matrix R and translation vector $\vec{t}$. The two basic steps of ICP can be summarized as follows:

Firstly, according to the (*k* - 1)th rigid transformation $\left(R_{k-1},{\vec{t}}_{k-1}\right)$, we set up correspondence between two point sets:

$$c_{k}(i)=\underset{c(i)\in \left\{1,2,\cdots ,N_{m}\right\}}{\arg\;\min}\|(R_{k-1}{\vec{p}}_{i}+{\vec{t}}_{k-1})-{\vec{m}}_{c(i)}{\|}_{2}^{2},i=1,2,\cdots ,N_{p}$$ (2)

Secondly, compute the new rigid transformation between ${\left\{R_{k-1}{\vec{p}}_{i}+{\vec{t}}_{k-1}\right\}}_{i=1}^{N_{p}}$ and ${\left\{{\vec{m}}_{c_{k}\left(i\right)}\right\}}_{i=1}^{N_{p}}$:

$$(R^{*},\vec{t^{*}})=\underset{R^{T}R=I_{n},\det(R)=1,\vec{t}}{\arg\;\min}\sum_{i=1}^{N_{p}}\|R(R_{k-1}{\vec{p}}_{i}+{\vec{t}}_{k-1})+\vec{t}-{\vec{m}}_{c_{k}(i)}{\|}_{2}^{2}$$ (3)

The ICP algorithm begins with an initial rigid transformation, and then it repeats the above two steps until convergence. However, it has been proved that this method is locally convergent, which means that the algorithm is easily failed when the rotation angle between two point sets is large. For this reason, a good initial transformation is so important that it guarantees that the algorithm converges to the global minimum finally.

## Our algorithm

In this section, we built a new objective function based on a global reference point, which is rotation invariant. After that, a new ICP algorithm is proposed to deal with this problem, which is demonstrated to be convergent.

### 3.1 Problem statement

The traditional ICP algorithm can register two point sets rapidly and precisely under the good initial values, but in practice, the initial rigid transformation is difficult to obtain. In this case, if the initial rotation angle is large and we still set up correspondence by finding the closest point, the original ICP algorithm will converge to a local minimum and the registration cannot be accomplished.

**Fig 1:**
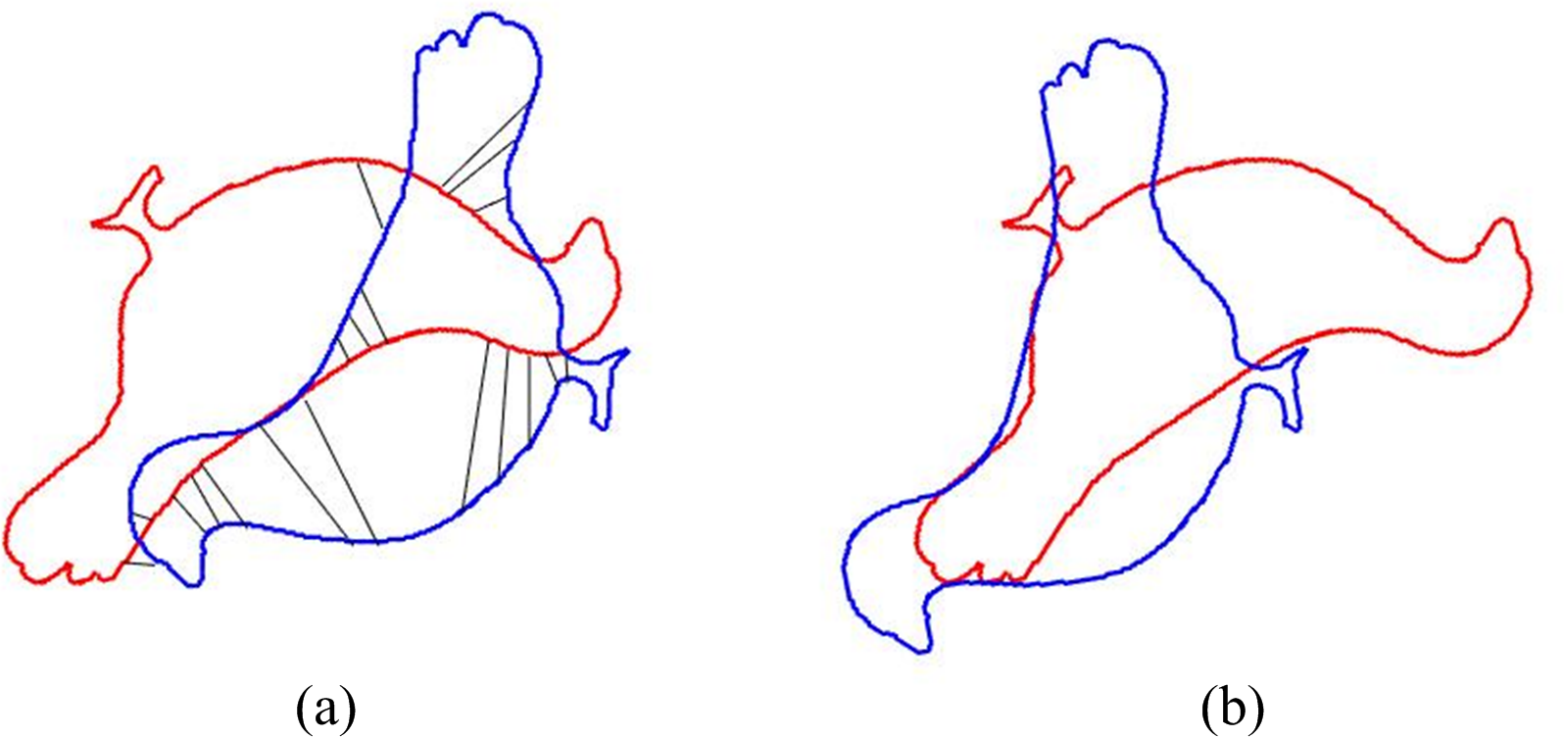
The registration of ICP. (a) Correspondence is established by searching closest points. (b) Registration results of ICP.

In Fig 1, the rotation angle between two point sets is large and correspondence is set up by searching the red points which are the closest to the blue point set. Obviously, the original ICP algorithm is totally wrong for this case. Although the error goes down, merely the two point sets become closer. Finally the ICP algorithm cannot register these two point sets well.

**Fig 2:**
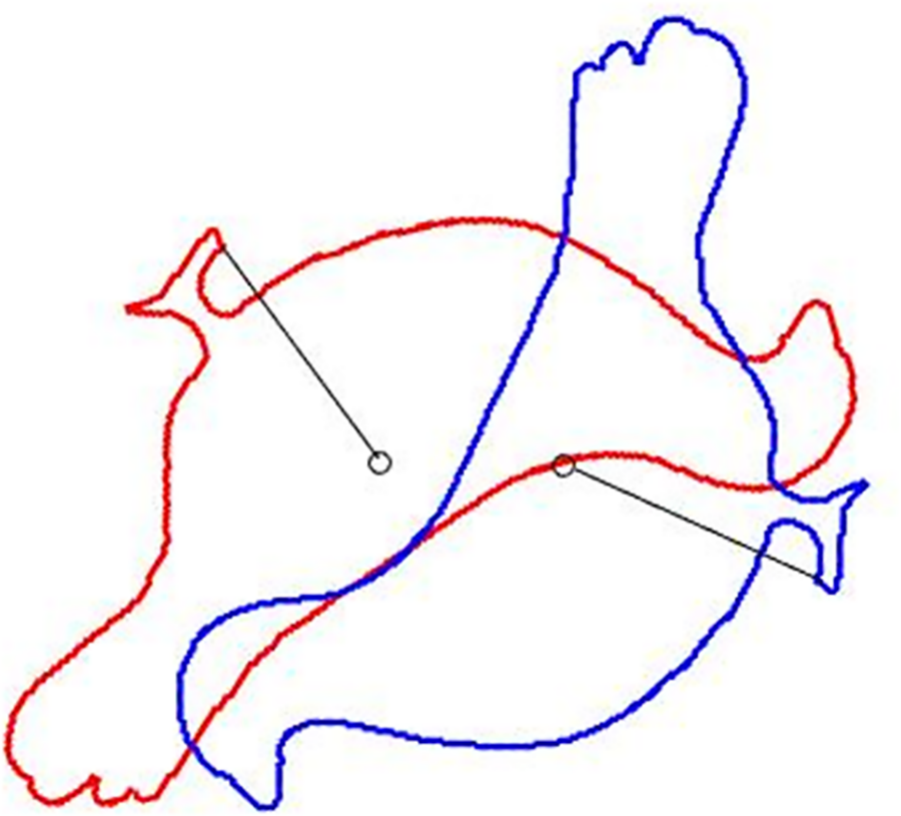
The global reference point and the rotation invariant feature.

According to the above statement, the ICP algorithm depends heavily on the initial rotation. Aiming at the problem like this, we need to find a rotation invariant measurement. With the aid of this invariant, correspondence is established correctly even the initial rigid transformation is wrong. Along with this idea, we introduce a global reference point, such as the mean point of the point set, and then compute the distance between each point of point sets and the global reference point, which is rotation invariant. In Fig 2, we take the mean point of the point sets as a global reference point. And the distance between each point of point sets and the global reference point will not change, no matter how the point set rotate or translate. By means of this trait, we can introduce this measurement into the LS problem to set up the objective function, which is given as follows.

Given two point sets in ℝ^*n*^, the shape point set $P≜{\left\{{\vec{p}}_{i}\right\}}_{i=1}^{N_{p}}(N_{p}\in N)$ and the model point set $M≜{\left\{{\vec{m}}_{j}\right\}}_{j=1}^{N_{m}}(N_{m}\in N)$. To set up the correspondence, we introduce the global reference points for two point sets respectively, which are ${\vec{p}}_{G}$ of P and ${\vec{m}}_{G}$ of M. For each point ${\vec{p}}_{i}={\left(x_{i}^{p},y_{i}^{p}\right)}^{T}$ of the shape point set P, we can calculate the Euclidean distance $d_{i}^{p}$ between ${\vec{p}}_{i}$ and ${\vec{p}}_{G}$. In the same way, the Euclidean distance $d_{j}^{m}$ between ${\vec{m}}_{j}={\left(x_{j}^{m},y_{j}^{m}\right)}^{T}$ and ${\vec{m}}_{G}$ is calculated. Because the original positions and the new distance are different measurements, we use *w* to be the weight to control the relative contribution of the positions and features. In this way, the goal of the registration is to establish the correspondence between these two sets via the rigid transformation, which can be expressed as the following LS problem:

$$\begin{array}{l} \underset{R,\vec{t},c(i)\in \left\{1,2,\cdots ,N_{m}\right\}}{\min}\sum_{i=1}^{N_{p}}\left(\|(R{\vec{p}}_{i}+\vec{t})-{\vec{m}}_{c(i)}{\|}_{2}^{2}+w{\left(d_{i}^{p}-d_{c(i)}^{m}\right)}^{2}\right)\\ \;s.t.\;R^{T}R=I_{n},\det(R)=1 \end{array}$$ (4)

The objective function is composed of two terms. The former term is the traditional Euclidean distance between two point sets and the latter one is to measure the distance of the rotation invariant term which will not be affected by rotation transformation.

### 3.2 The robust ICP algorithm based on global reference point

Using the framework of the standard ICP algorithm, we give a variant of the ICP algorithm to solve the problem (4). As we introduce a global reference point, and the distance between the each point of sets and the global reference point is the rotation invariant feature, accordingly the new algorithm can accomplish the rigid registration whatever the rotation angle is. Similar to the ICP algorithm, the detailed steps of the algorithm proposed are present as follows.

Given the global reference points for two point sets respectively, such as the mean point of the sets, ${\vec{p}}_{G}$ of P and ${\vec{m}}_{G}$ of M. For each point of the point sets, calculate the Euclidean distance between themselves and the global reference point:

$$\left\{ \begin{array}{l} d_{i}^{p}=\|{\vec{p}}_{i}-{\vec{p}}_{G}{\|}_{2},i=1,2,\cdots ,N_{p}\\ d_{j}^{m}=\|{\vec{m}}_{j}-{\vec{m}}_{G}{\|}_{2},j=1,2,\cdots ,N_{m} \end{array}\right.$$ (5)

Moreover, we give the weight *w*, and then the algorithm can be accomplished by repeating the following two steps using the distance mentioned above.

Firstly, we set up the correspondence by the (*k* - 1)th rigid transformation $\left(R_{k-1},{\vec{t}}_{k-1}\right)$:

$$c_{k}(i)=\underset{c(i)\in \{1,2,\cdots N_{m}\}}{\arg\;\min}(\|(R_{k-1}{\vec{p}}_{i}+{\vec{t}}_{k-1})-{\vec{m}}_{c(i)}{\|}_{2}^{2}+w{\left(d_{i}^{p}-d_{c(i)}^{m}\right)}^{2}),i=1,2,\cdots ,N_{p}$$ (6)

In the proposed algorithm, the process of setting up the correspondence between two point sets is similar to the standard ICP algorithm. In Eq (6), the former term is the Euclidean distance between two point sets and we add the latter term to extend the original vector. For the original points ${\vec{p}}_{i}$ and ${\vec{m}}_{j}={\left(x_{j}^{m},y_{j}^{m}\right)}^{T}$, the vector could be extended to ${\vec{x}}_{i}={\left(R{\vec{p}}_{i}+\vec{t},wd_{i}^{p}\right)}^{T}$ and ${\vec{y}}_{c(i)}={\left({\vec{m}}_{c(i)},wd_{c(i)}^{m}\right)}^{T}$. Therefore, Eq (6) is rewritten as the following function.

$$c_{k}(i)=\underset{c(i)\in \{1,2,\cdots N_{m}\}}{\arg\;\min}\|{\vec{x}}_{i}-{\vec{y}}_{c(i)}{\|}_{2}^{2},i=1,2,\cdots ,N_{p}$$ (7)

In the above equation, we search the closest point in (*n*+1)-dimension space to establish a more accurate correspondence between two point sets. This step can be solved by many methods such as *k*-d tree [17] and Delaunay tessellation [18,19]. In this paper, we choose the latter to search the nearest point.

Secondly, we calculate the new rigid transformation via the known correspondence of the *k*th step. In Eq (4), the former term is the same as the traditional ICP and the latter one is a rotation invariant, which won’t change through the rigid transformation. The latter term has no impact on the transformation, but it can be helpful for establishing the correspondence. Therefore, the new rigid transformation is calculated by the following term:

$$(R_{k},{\vec{t}}_{k})=\underset{R^{T}R=I_{n},\det(R)=1,\vec{t}}{\arg\;\min}\sum_{i=1}^{N_{p}}\|R{\vec{p}}_{i}+\vec{t}-{\vec{m}}_{c_{k}(i)}{\|}_{2}^{2}$$ (8)

The method of calculating the rigid transformation is the same as the original ICP algorithm. This problem can be solved by singular value decomposition (SVD) [20]. The detailed procedures can be summarized as follows:

$$H=\sum_{i=1}^{N_{p}}({\vec{p}}_{i}-{\vec{p}}_{G}){\left({\vec{m}}_{c(i)}-{\vec{m}}_{G}\right)}^{T}$$

where the singular value decomposition of H is:

$$H=U\Lambda V^{T}$$

Therefore, the rotation matrix is computed as follows:

$$R_{k}=VU^{T}$$ (9)

Moreover, the translation vector is calculated as the following equation:

$${\vec{t}}_{k}={\vec{m}}_{G}-R_{k}{\vec{p}}_{G}$$ (10)

This new algorithm repeats the above two steps until convergence. In the iterative procedures, the weight *w* is non-increasing, which can make the algorithm much more precise. To control the convergence of algorithm via the change of the weight *w*, we give a threshold *T*. When the root mean square (RMS) error between two point sets is smaller than *T*, we set the weight to be RMS divides by a preset number *a*, where we set this number to be 20 in this paper.

Hence, we outline the proposed algorithm as follows.

Input: Two point sets $P≜{\left\{{\vec{p}}_{i}\right\}}_{i=1}^{N_{p}}(N_{p}\in N)$ and $M≜{\left\{{\vec{m}}_{j}\right\}}_{j=1}^{N_{m}}(N_{m}\in N)$.

Initialize: Rotation matrix R_0_, translation vector ${\vec{t}}_{0}$, weight *w*_0_, threshold *T* and a small enough number *ε*.

Repeat

Step 1: Compute (7) to set up correspondence by the (*k* - 1)th transformation $\left(R_{k-1},{\vec{t}}_{k-1}\right)$.

Step 2: Compute (8) to obtain the new transformation $\left(R_{k},{\vec{t}}_{k}\right)$ via Eqs (10) and (11).

Step 3:

If RMS_*k*_ < *T*, then *w*_*k*_ = RMS_*k*_/*a*.
Else *w*_*k*_ = *w*_*k*−1_

Until |RMS_*k*_ − RMS_*k*−1_| < *ε* or *k* reaches a maximum number of iterations.

Output: Rotation matrix R_*k*_ and translation vector ${\vec{t}}_{k}$.

### 3.3 Theory analysis

In this section, the convergence property of our algorithm is analyzed. In addition, as the importance of the weight *w* in the whole algorithm, the selection principle and the adjustment strategy of *w* are discussed.

#### 3.3.1 Convergence theorem

As the procedure of the proposed ICP algorithm is similar to that of the original ICP algorithm, we can demonstrate that it is a local convergent algorithm.

Theorem 1. The proposed algorithm converges monotonically to a local minimum with respect to square distance.

Proof. Given shape point set $P≜{\left\{{\vec{p}}_{i}\right\}}_{i=1}^{N_{p}}(N_{p}\in N)$ and Fmodel point set $M≜{\left\{{\vec{m}}_{j}\right\}}_{j=1}^{N_{m}}(N_{m}\in N)$. Let $q_{i,k\text{-}1}≜R_{k\text{-}1}{\vec{p}}_{i}+{\vec{t}}_{k\text{-}1}$, where R_*k*−1_ is a rotation matrix, and ${\vec{t}}_{k-1}$ is a translation vector. The correspondence can be established by (6), and the Euclidean distance with weighted rotation invariant feature shall be denoted as:

$$d_{w}({\vec{p}}_{i},{\vec{m}}_{j})=d_{e}({\vec{p}}_{i},{\vec{m}}_{j})+wd_{f}({\vec{p}}_{i},{\vec{m}}_{j})$$

where $d_{e}({\vec{p}}_{i},{\vec{m}}_{j})=\|{\vec{p}}_{i}-{\vec{m}}_{j}{\|}_{2}^{2}$, $d_{f}({\vec{p}}_{i},{\vec{m}}_{j})={\left(d_{i}^{p}-d_{j}^{m}\right)}^{2}$.

In the (*k*−1)th step, the square distance is:

$$e_{k-1}=\sum_{i=1}^{N_{p}}\left[d_{e}({\vec{q}}_{i,k-1},{\vec{m}}_{c_{k-1}(i)})+w_{k-1}^{}d_{f}({\vec{q}}_{i,k-1},{\vec{m}}_{c_{k-1}(i)})\right]$$

After the rotation R_*k*_ and ${\vec{t}}_{k}$ of Eq (9) are computed, the error becomes:

$${\tilde{e}}_{k-1}=\sum_{i=1}^{N_{p}}\left[d_{e}({\vec{q}}_{i,k},{\vec{m}}_{c_{k-1}(i)})+w_{k-1}^{}d_{f}({\vec{q}}_{i,k},{\vec{m}}_{c_{k-1}(i)})\right]$$

As the transformation R_*k*_ and ${\vec{t}}_{k}$ minimizes the error between two point sets, we obtain:

$$\sum_{i=1}^{N_{p}}d_{e}({\vec{q}}_{i,k},{\vec{m}}_{c_{k-1}(i)})\leq \sum_{i=1}^{N_{p}}d_{e}({\vec{q}}_{i,k-1},{\vec{m}}_{c_{k-1}(i)})$$

Because the invariant term does not change with rigid transformation,

$$\sum_{i=1}^{N_{p}}w_{k-1}^{}d_{f}({\vec{q}}_{i,k},{\vec{m}}_{c_{k-1}\left(i\right)})=\sum_{i=1}^{N_{p}}w_{k-1}^{}d_{f}({\vec{q}}_{i,k-1},{\vec{m}}_{c_{k-1}\left(i\right)})$$

we have ${\tilde{e}}_{k-1}\leq e_{k-1}$.

Next, the weight is updated to be *w*_*k*_. We set the square error as:

$$\delta_{k}=\sum_{i=1}^{N_{p}}\left[d_{e}({\vec{q}}_{i,k},{\vec{m}}_{c_{k-1}(i)})+w_{k}d_{f}({\vec{q}}_{i,k},{\vec{m}}_{c_{k-1}(i)})\right]$$

As the weight *w* is non-increasing, we get:

$$\sum_{i=1}^{N_{p}}w_{k}^{}d_{f}({\vec{q}}_{i,k},{\vec{m}}_{c_{k-1}\left(i\right)})\leq \sum_{i=1}^{N_{p}}w_{k-1}^{}d_{f}({\vec{q}}_{i,k},{\vec{m}}_{c_{k-1}\left(i\right)})$$

Therefore,

$$\delta_{k}\leq {\tilde{e}}_{k-1}$$

In the *k*th step, the square error is:

$$e_{k}=\sum_{i=1}^{N_{p}}\left[d_{e}({\vec{q}}_{i,k},{\vec{m}}_{c_{k}(i)})+w_{k}^{}d_{f}({\vec{q}}_{i,k},{\vec{m}}_{c_{k}(i)})\right]$$

According to the closest point principle,

$$e_{k}\leq \delta_{k}$$

Hence, repeat procedures above, we obtain:

$$0\leq \cdots \leq e_{k}\leq \delta_{k}\leq {\tilde{e}}_{k-1}\leq e_{k-1}\leq \cdots ,\;\mathrm{for}\;\mathrm{all}\;k.$$

As our algorithm converges locally, a good initial transformation is of great importance. A rotation invariant term is introduced to deal with the problem of the initial values, which will be discussed in the next section.

#### 3.3.2 The rotation invariant weight

As the proposed algorithm is a local convergent algorithm, the rotation invariant term is introduced to tackle this problem. In our algorithm, the weight *w* is of great importance. When the weight of the rotation invariant term is larger, the objective function (4) is much more robust to the rotation. Otherwise, the objective function is much more focus on the registration precision. Specially, our algorithm will degenerate to the traditional ICP when *w* equals to zero.

**Fig 3:**
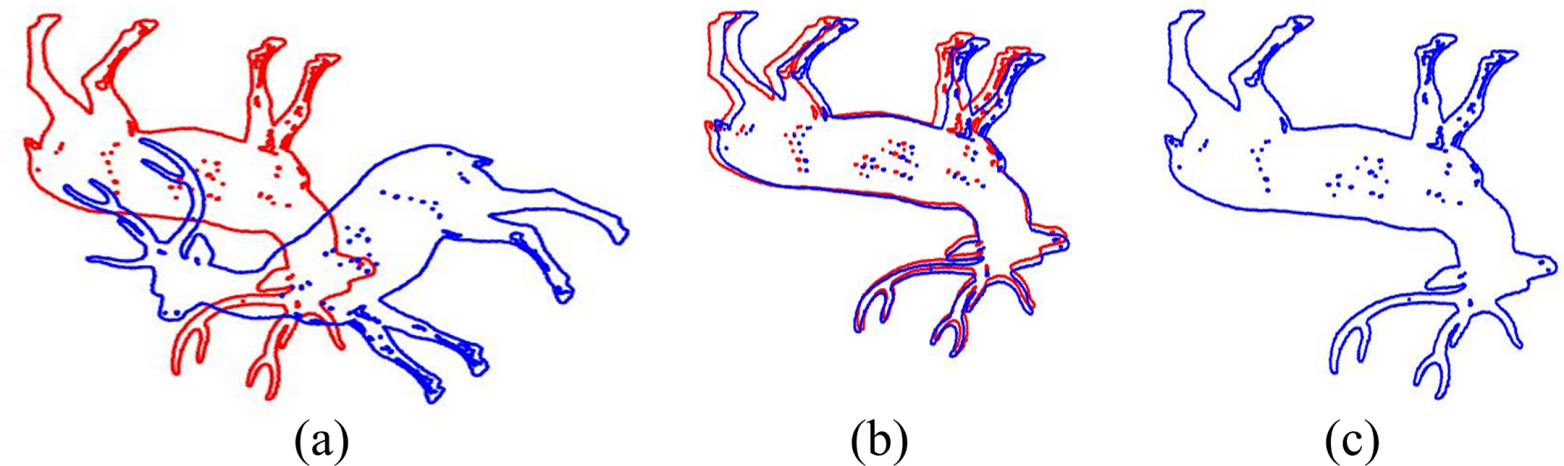
A registration example. (a) Point sets before registration. (b) The intermediate result of our algorithm when the weight is large. (c) Final registration result of our algorithm when the weight is quite small.

In the initialization of our algorithm, a quite large number is given for the weight *w*. During iterations, the error between the transformed point set and the model point set is gradually decreasing, which means the shape point set is transformed to the correct direction guided by the rotation term. In Fig 3(B), it is obviously that a large weight can guide the shape point set to a relatively correct position. In this time, the error is small and it means that the rotation matrix is approaching to the ground-truth. On this occasion, the former term of the objective function (4), which describes the precision of registration, should be much accounted of rather than the latter one which is a rotation invariant. Therefore, we make *w* go down as the error reduces to accomplish the accurate registration. In this way, a coarse-to-fine method is used to register two point sets with large rotation angle. The final result of the registration is shown in Fig 3(C).

## Experimental results

To prove the robustness, accuracy and convergence of our algorithm, we conduct the experiments on simulation and standard data of MPEG-7 CE-Shape-1 dataset [21], where our algorithm is compared with the original ICP.

### 4.1 2D simulations

In this section, the 2D Bird, Cup and Deer data from S1 Data are chosen as shape data to conduct the experiments. To demonstrate that our algorithm can deal with the registration for large-angle rotation, we carry out six experiments of six different rotation angles. To obtain the model point sets, we rotate the shape point sets with angle 30°, 60°, 90°, 120°, 150° and 180° respectively and translate the data randomly.

To compare the proposed algorithm with ICP, we define the *ε*_R_ = ‖R_*t*_ − R‖_2_, $\varepsilon_{\vec{t}}=\|{\vec{t}}_{t}-\vec{t}{\|}_{2}$. We transform the original data by R_*t*_ and ${\vec{t}}_{t}$, and compute the R and $\vec{t}$ by the registration algorithm. In addition, the root mean square (RMS) is used to measure these two algorithms, and all the results are presented in Table 1.

**Table 1:** Comparison of 2D simulation results.

Point sets		Angle	30	60	90	120	150	180
**Bird**	ICP	*ε*_R_	2.58e-15	2.69e-15	2.65	2.65	2.66	2.66
		$\varepsilon_{\vec{t}}$	1.99e-13	3.37e-13	439.23	439.23	439.78	439.77
		RMS	1.71e-13	1.75e-13	25.70	25.70	25.70	25.70
	Our Algorithm	*ε*_R_	1.24e-16	3.33e-16	2.33e-16	1.57e-16	1.19e-15	4.41e-16
		$\varepsilon_{\vec{t}}$	2.93e-14	1.76e-13	9.94e-14	1.21e-13	2.05e-13	1.14e-13
		RMS	4.53e-14	1.13e-13	1.18e-13	1.34e-13	9.73e-14	1.65e-13
**Cup**	ICP	*ε*_R_	1.26e-15	0.02	2.62	2.70	2.75	2.81
		$\varepsilon_{\vec{t}}$	1.42e-13	4.23	507.74	521.73	529.60	539.08
		RMS	2.50e-13	1.41	22.81	21.97	22.17	23.00
	Our Algorithm	*ε*_R_	6.47e-16	3.55e-16	6.67e-16	6.93e-16	3.85e-16	4.51e-16
		$\varepsilon_{\vec{t}}$	5.73e-14	1.21e-13	8.53e-14	7.11e-14	3.38e-13	3.60e-13
		RMS	1.22e-13	5.04e-14	7.69e-14	1.19e-13	4.19e-13	2.63e-13
**Deer**	ICP	*ε*_R_	9.31e-07	8.69e-16	2.11	2.77	2.77	2.83
		$\varepsilon_{\vec{t}}$	0.85	2.41e-13	847.89	1.14e+03	1.14e+03	1.11e+03
		RMS	0.69	3.34e-13	59.12	40.01	40.01	43.21
	Our Algorithm	*ε*_R_	6.40e-16	7.11e-16	2.00e-15	8.62e-16	6.21e-16	1.84e-05
		$\varepsilon_{\vec{t}}$	7.13e-13	1.21e-13	1.63e-12	3.41e-13	3.22e-13	0.86
		RMS	9.87e-13	1.64e-13	1.21e-12	4.17e-13	1.80e-13	0.69

From Table 1, it can be seen that our algorithm is more robust than ICP. Specifically, when the rotation angle is 30° or 60°, *ε*_R_, $\varepsilon_{\vec{t}}$ and RMS of our algorithm are similar to ICP, which means that these two algorithms can complete the registration while the rotation angle between two point sets is small. Moreover, our algorithm is more accurate. However, when the rotation angle between two point sets is large such as 90°, 120°, 150° and 180°, the original ICP could not accomplish all the point sets registration, while our algorithm does well in all the rotation angles between two point sets, which proves the robustness of our algorithm.

**Fig 4:**
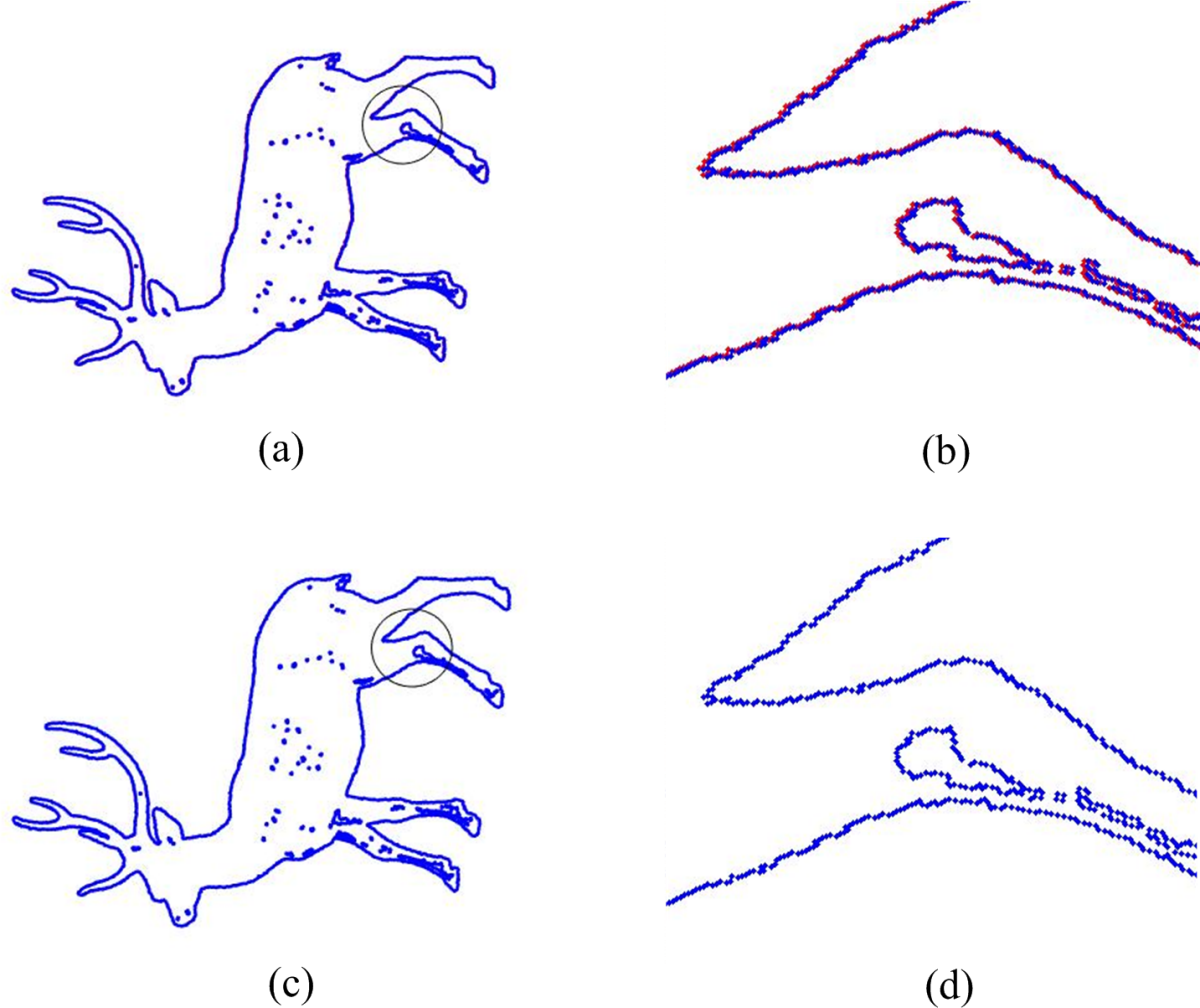
Local amplification registration results for Deer in which rotation angle is 30°. (a) Registration result of ICP. (b) Local amplification result of ICP. (c) Registration result of our algorithm. (d) Local amplification result of our algorithm.

To demonstrate the accuracy of our algorithm intuitively, the local amplification results of the point sets with 30° rotation angle are shown in Fig 4. Red points represent the model point sets, and blue points represent the shape point sets after rigid transformation. It can be clearly seen that the result of our algorithm is more accurate than the original ICP.

**Fig 5:**
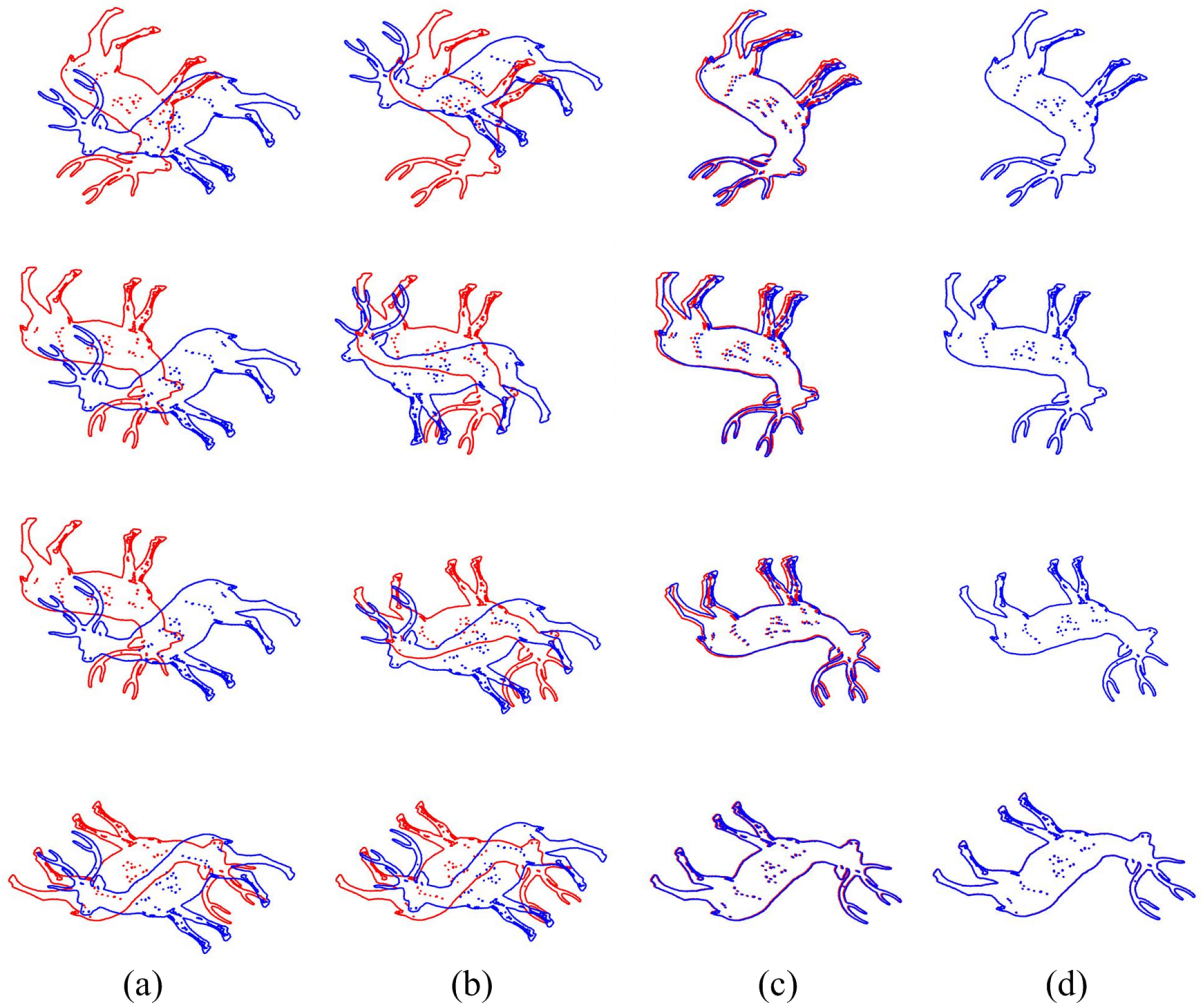
Comparison of two registration algorithms for Deer in which rotation angles are 90°, 120°, 150° and 180° (from up to down). (a) Point sets before registration. (b) Registration results of ICP. (c) The intermediate results of our algorithm when the weight of the rotation invariant is large. (d) Registration results of our algorithm.

As for initial rotation angle between two point sets is large, Fig 5 displays the comparison of the original ICP and the proposed algorithm. The distance between two point sets goes to smaller slightly by ICP, and the registration fails ultimately. However, our algorithm can amend the large-angle rotation between two point sets by using a rotation invariant. As the intermediate results show, our algorithm rotate the shape point sets from a relatively large angle to a small angle. On this basis, the registration completes finally.

**Fig 6:**
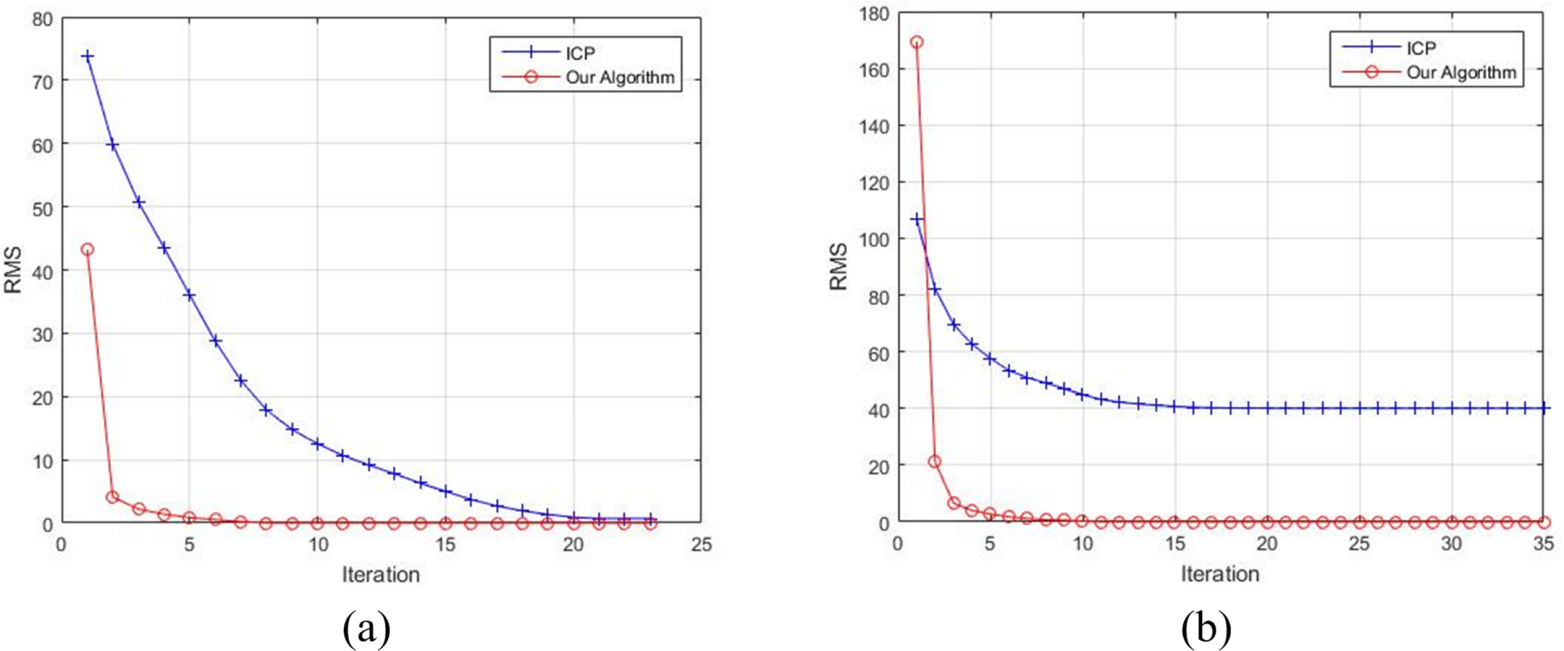
The convergence of ICP and our algorithm for Deer. (a) Deer with 30° rotation. (b) Deer with 120° rotation.

Moreover, the comparison of the convergence of our algorithm and ICP with respect to different rotation degrees is shown in Fig 6. On the one hand, when the rotation angle between two point sets is small, the RMS of ICP approaches to zero gradually via multiple iterative steps. By contrast, the proposed algorithm needs a few iterative steps to complete the registration. On the other hand, when the rotation angle between two point sets is large, apparently ICP would trap in a local minimum value and fail to register the two point sets. However, our algorithm can deal with this problem well by means of the rotation invariance. Meanwhile, the RMS of our algorithm decreases rapidly after a few steps and is close to zero finally. In a word, our algorithm converges with fewer steps whatever the initial rotation angle between two point sets.

In the simulation experiments, the accuracy, convergence and robustness of our algorithm have been proved. Whatever the rotation angle between two point sets is, the algorithm we proposed can complete the registration efficiently and precisely.

#### 4.2 2D standard database

**Fig 7:**
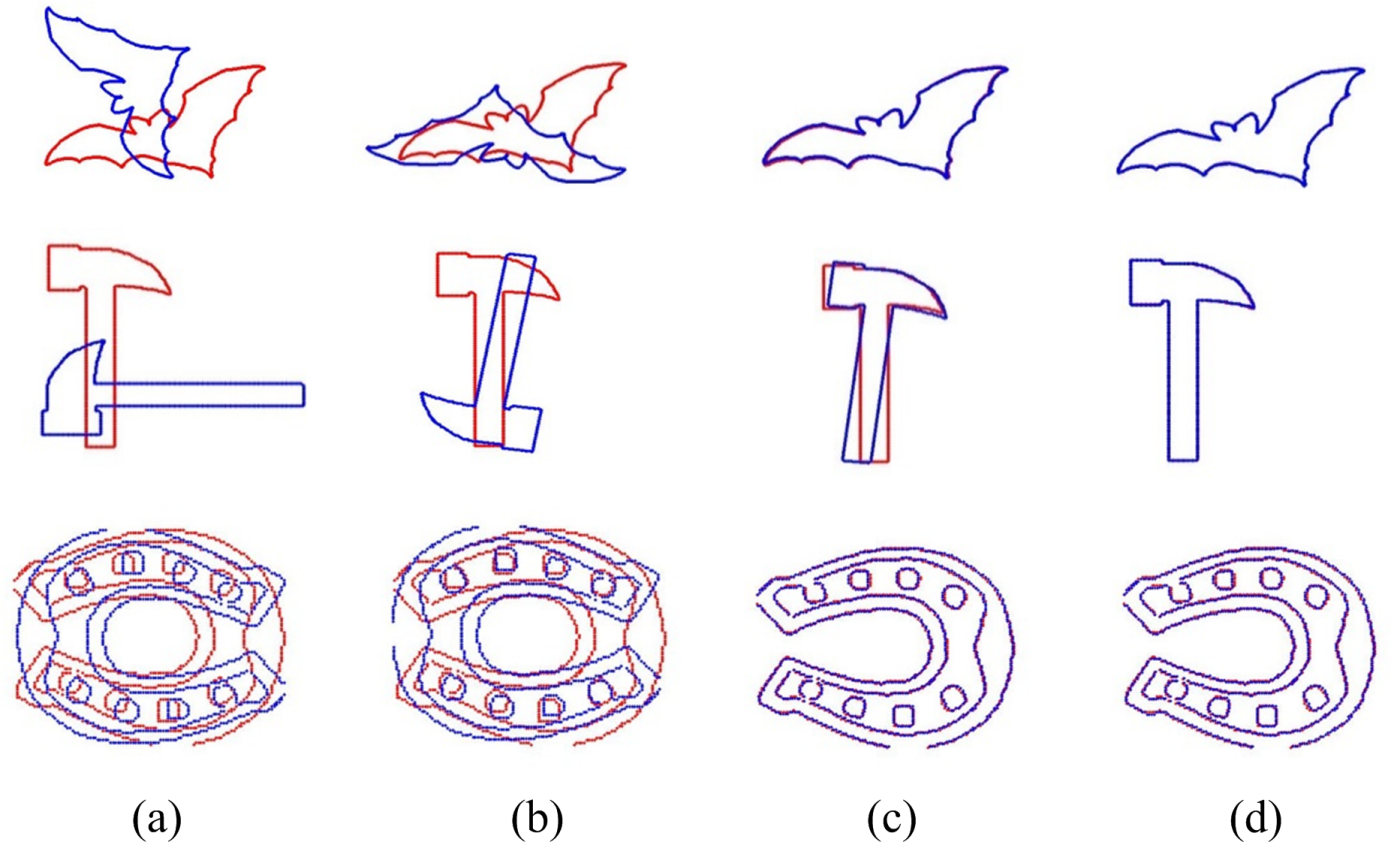
Comparison of two registration algorithms for Bat, Hammer and Horseshoe (from up to down). (a) Point sets before registration. (b) Registration results of ICP. (c) The intermediate results of our algorithm when the weight is large. (d) Registration results of our algorithm.

In this part, we compared these two algorithm on the part B of CE-Shape-1 database. In the experiments, three couples of Bat, Hammer and Horseshoe from S1 Data are used for registration. Moreover, the RMS errors of these two algorithms are given in Table 2, while *ε*_R_ and $\varepsilon_{\vec{t}}$ cannot be computed as the ground-truth of the rigid transformation could not be obtained. The registration results of these two algorithms are shown in Fig 7.

**Table 2:** RMS comparison of 2D point sets.

Point Sets	ICP	Our Algorithm
**Bat**	50.30	0.43
**Hammer**	17.09	0.31
**Horseshoe**	3.01	0.56

**Fig 8:**
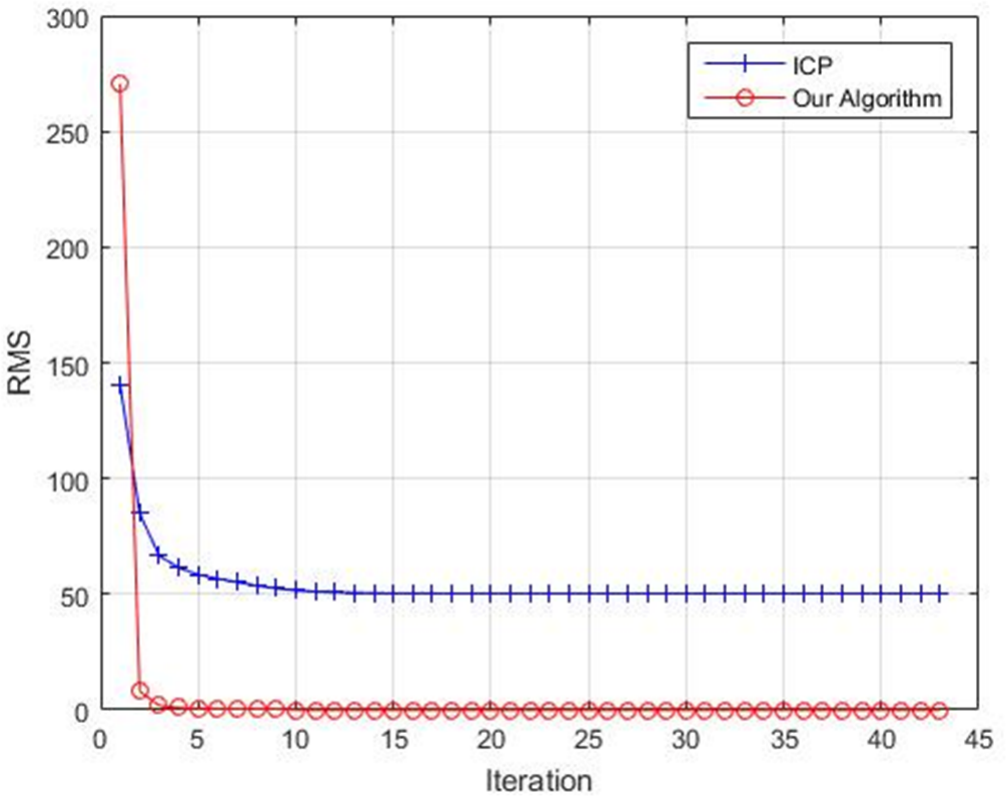
The convergence of ICP and our algorithm for Bat.

It can be seen in Table 2 that RMS of our algorithm is smaller than ICP. In Fig 7, it is obvious that ICP fails to register the two point sets but our algorithm does well in registering the point sets with large rotation angle. Meanwhile, Fig 8 gives the convergence comparison for these two algorithms. Our algorithm converges with fewer steps than ICP and the RMS of our algorithm is close to zero finally.

In conclusion, the proposed algorithm for 2D standard database is robust and convergent. Moreover, fewer steps of iterations are required to complete the registration.

## Conclusion

This paper proposes a variant of ICP algorithm based on global reference point to deal with the problem of registering two point sets with large rotation angle. Aiming at solving this problem, a global reference point is introduced and the distance between each point and global reference point is rotation invariant. Hence a more accurate correspondence is established, which provides a good initial for registration. Then the registration can be accomplished efficiently and accurately.

Compared to the previous works, our contributions include two main aspects: 1) To avoid the traditional ICP algorithm being trapped into the local minimum, a global reference point is introduced and the more accurate correspondence is set up by the rotation invariance. 2) Fewer steps of iterations are required to complete the registration. Therefore, the proposed algorithm is faster and some experimental results demonstrate that our algorithm performs better than ICP.

Even though the proposed method can solve the rotation invariant registration, there are still many problems to be dealt with. In the future, our algorithm can be extended to registration for 3D data and some applications, such as medical image registration.

## Supporting information

S1 Data The minimal data set. (ZIP)
