# Supplementary figures and images for: Robust iterative closest point algorithm based on global reference point for rotation invariant registration
Source: PLoS One. 2017 Nov 27;12(11):e0188039. doi: 10.1371/journal.pone.0188039 (PMC5703502; doi:10.1371/journal.pone.0188039)

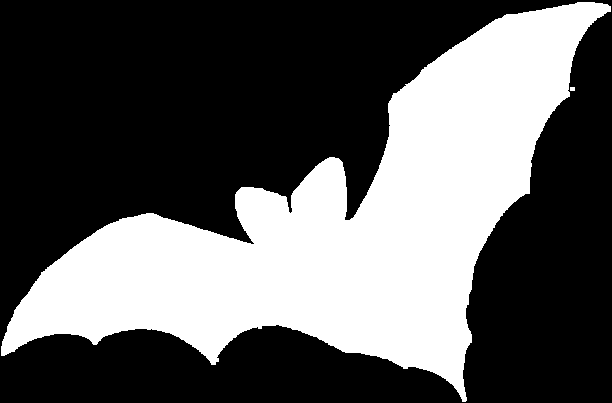

Supplement: S1 Data — (ZIP) [file pone.0188039.s001.zip › bat-18.gif]

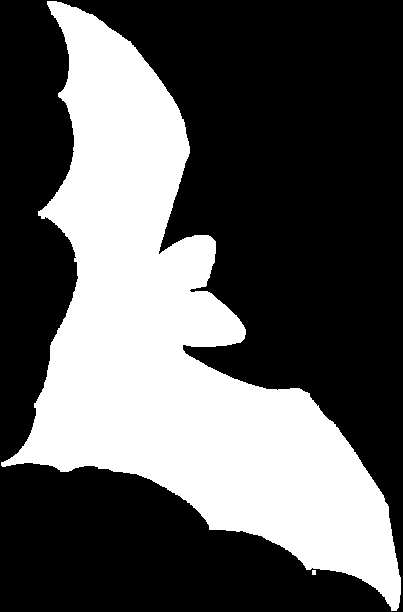

Supplement: S1 Data — (ZIP) [file pone.0188039.s001.zip › bat-19.gif]

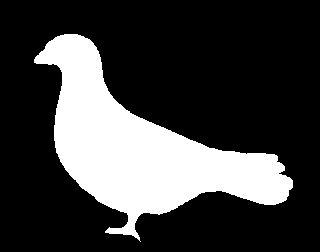

Supplement: S1 Data — (ZIP) [file pone.0188039.s001.zip › bird-17.gif]

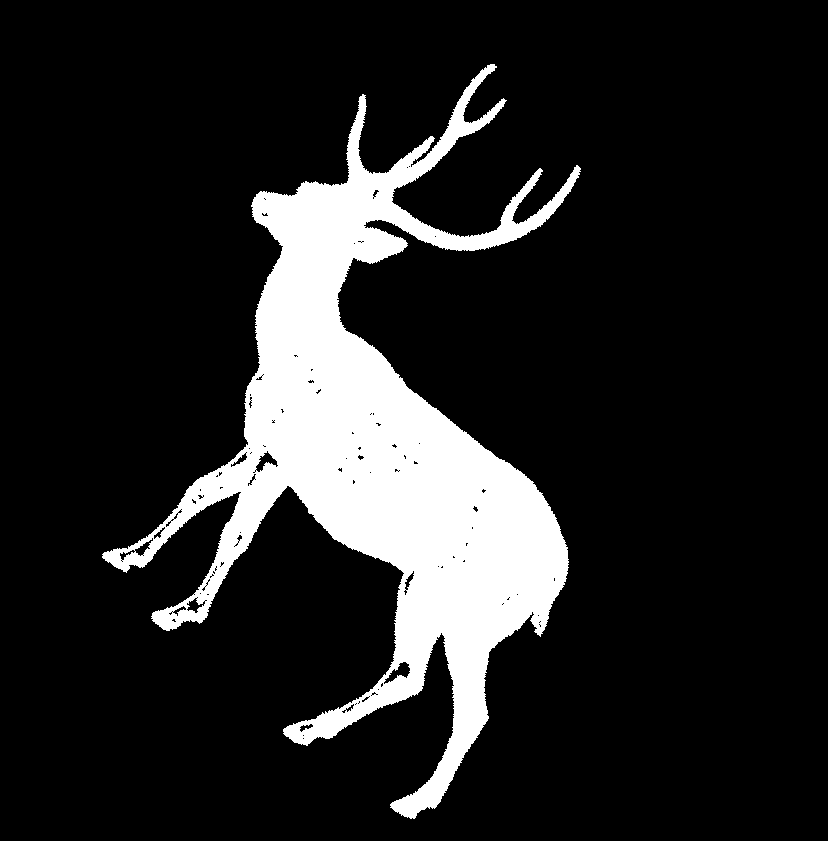

Supplement: S1 Data — (ZIP) [file pone.0188039.s001.zip › deer-17.gif]

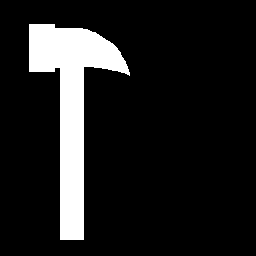

Supplement: S1 Data — (ZIP) [file pone.0188039.s001.zip › hammer-4.gif]

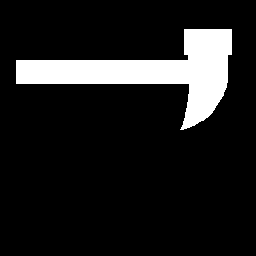

Supplement: S1 Data — (ZIP) [file pone.0188039.s001.zip › hammer-5.gif]

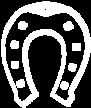

Supplement: S1 Data — (ZIP) [file pone.0188039.s001.zip › horseshoe-11.gif]

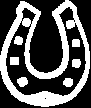

Supplement: S1 Data — (ZIP) [file pone.0188039.s001.zip › horseshoe-19.gif]
